# The Nucleoid-Associated Protein GapR Uses Conserved Structural Elements To Oligomerize and Bind DNA

**DOI:** 10.1128/mBio.00448-20

**Published:** 2020-06-09

**Authors:** Rogério F. Lourenço, Saumya Saurabh, Jonathan Herrmann, Soichi Wakatsuki, Lucy Shapiro

**Affiliations:** a Department of Developmental Biology, Stanford University School of Medicine, Stanford, California, USA; b Department of Structural Biology, Stanford University School of Medicine, Stanford, California, USA; c Bioscience Division, SLAC National Accelerator Laboratory, Menlo Park, California, USA; d Chan Zuckerberg Biohub, San Francisco, California, USA; University of Washington

**Keywords:** nucleoid-associated protein, oligomeric state, DNA binding, structure/function conservation

## Abstract

Nucleoid-associated proteins (NAPs) are DNA binding proteins critical for the organization and function of the bacterial chromosome. A newly discovered NAP in Caulobacter crescentus, GapR, is thought to facilitate the movement of the replication and transcription machines along the chromosome by stimulating type II topoisomerases to remove positive supercoiling. Here, utilizing genetic, biochemical, and biophysical studies of GapR in light of a recently published DNA-bound crystal structure of GapR, we identified the structural elements involved in oligomerization and DNA binding. Moreover, we show that GapR is maintained as a tetramer upon its dissociation from DNA and that tetrameric GapR is capable of binding DNA molecules *in vitro*. Analysis of protein chimeras revealed that two helices of GapR are functionally conserved in H-NS, demonstrating that two evolutionarily distant NAPs with distinct mechanisms of action utilize conserved structural elements to oligomerize and bind DNA.

## Abstract (precis)

Bacteria organize their genetic material in a structure called the nucleoid, which needs to be compact to fit inside the cell and, at the same time, dynamic to allow high rates of replication and transcription. Nucleoid-associated proteins (NAPs) play a pivotal role in this process, so their detailed characterization is crucial for our understanding of DNA organization into bacterial cells. Even though NAPs affect DNA-related processes differently, all of them have to oligomerize and bind DNA for their function. The significance of this study is the identification of structural elements involved in the oligomerization and DNA binding of a newly discovered NAP in C. crescentus and the demonstration that structural elements are conserved in evolutionarily distant and functionally distinct NAPs.

## INTRODUCTION

Bacterial cells organize their genetic material in a compact and dynamic structure, the nucleoid, using DNA binding proteins referred to as nucleoid-associated proteins (NAPs) (1). GapR is a newly discovered NAP in Caulobacter crescentus, with orthologous proteins widespread in alphaproteobacteria (2–5). Either deleting or depleting *gapR* has been associated with cell division-related morphological changes (2, 4) and defects during DNA replication (3, 5) and chromosome segregation (5). By associating with overtwisted regions at the 3′ ends of highly transcribed genes and ahead of the replication fork, and stimulating type II topoisomerases to relax positive supercoiling, GapR was proposed to stimulate transcription and replication (3).

High-resolution crystal structures of DNA-bound GapR revealed a tetrameric protein assembly encircling DNA (3, 6). In all these structures, each subunit of GapR folds into three α-helices (H1, H2, and H3), all of them potentially involved in self-association: H1 yielding a dimer, and an interface formed by H2 and H3 promoting the dimer of dimers (3, 6). Moreover, the side chains of positively charged residues at H1 and H2 are pointed toward the central channel of the tetramer (3, 6) and are in close proximity to phosphate groups of the encircled DNA (3), suggesting their involvement in DNA binding. Despite the overall folding, the crystal structures slightly deviate from each other with respect to the position of H3, allowing the central channel to accommodate overtwisted or B-DNA (6). This plasticity in the tetramer structure is thought to be important for GapR to translocate along B-DNA, searching for overtwisted regions, and to form a tight complex at overtwisted DNA, where GapR stimulates topoisomerases (6).

In this study, we sought to investigate the structure-function relationship of C. crescentus GapR in oligomer formation and DNA binding. We showed that GapR maintains its tetrameric state even in the absence of DNA. *In vitro* and *in vivo* analyses of mutant GapR proteins led to the demonstration that the H1 and H3 helices are both critical for the assembly of GapR into the tetrameric structure while H2 is needed for DNA binding. Moreover, GapR is capable of bridging DNA molecules *in vitro*. By engineering chimeric proteins, we showed that two GapR structural elements are functionally conserved in the mechanistically distinct nucleoid-associated protein H-NS.

## RESULTS

### GapR remains a tetramer upon its dissociation from DNA.

**FIG 1:**
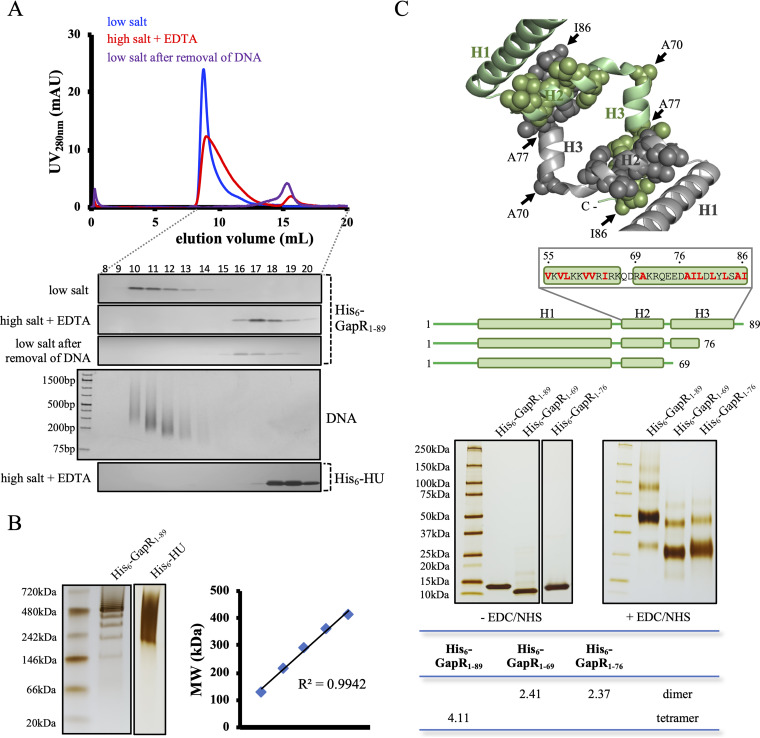
GapR isolated from copurified DNA is a tetramer, but mutant proteins with helix H3 deleted are dimers. (A) Analysis of the full-length GapR by size exclusion chromatography. His_6_-GapR_1–89_ purified without nuclease treatment was dialyzed against buffer containing either 150 mM NaCl (low salt) or 1 M NaCl supplemented with 1 mM EDTA (high salt plus EDTA) and analyzed by size exclusion chromatography using the Superdex 200 10/300 GL column. His_6_-GapR_1–89_ separated from copurified DNA was also analyzed by size exclusion chromatography (low salt after removal of DNA). Protein at 50 μM (calculated from the monomeric state) was used for all runs. Fractions 8 to 20 were resolved by SDS-PAGE, and the gels were silver stained. Shown below is a gel used to detect DNA prepared with fractions collected from protein treated under high-salt plus EDTA conditions and stained with ethidium bromide. His_6_-HU purified without nuclease treatment and dialyzed against high-salt plus EDTA buffer was used as a control. (B) Analysis of DNA-bound GapR by native PAGE. His_6_-GapR_1–89_ purified without nuclease treatment and dialyzed against low-salt buffer was resolved by PAGE under native conditions, and the gel was silver stained. The molecular weights calculated for a few bands of DNA-bound GapR are shown on the right. His_6_-HU under the same conditions was used as a control. (C) Determination of the oligomerization state of WT GapR and two truncated GapR proteins. The upper panel shows a structural representation of the interface involved in GapR tetramerization, derived from the DNA-bound crystal structure (PDB 6CG8) (3). GapR subunits are differentiated on the basis of color (gray and green), and hydrophobic residues in H2 and H3 are shown as spheres. Below is a schematic of full-length GapR and the two GapR truncation mutants, highlighting in red the hydrophobic residues in the H2 and H3 sequence. The lower panel shows SDS-PAGE of cross-linking assays performed with the proteins shown in the schematic. GapR proteins at 50 μM (monomer) were treated with cross-linking agents (400 mM EDC plus 100 mM NHS) for 2 h at room temperature, the reaction products were resolved by SDS-PAGE, and the gel was silver stained. EDC and HNS act by a two-step reaction to cross-link glutamic and aspartic acid to lysine residues (32). Reactions were conducted in the presence of 1 M NaCl and 1 mM EDTA as His_6_-GapR_1–69_ and His_6_-GapR_1–76_ precipitate in the presence of 150 mM NaCl. Under these conditions, GapR is not associated with DNA, ruling out the possibility that DNA could affect the oligomeric state of the proteins. As a control, proteins incubated for 2 h at room temperature in the absence of the cross-linking agents EDC and NHS were resolved by SDS-PAGE. The panel below the gel lists the apparent oligomeric state of each major band.

GapR, purified as a dimer, was found to assemble into a tetramer in the presence of DNA (3). To investigate the role of DNA on the oligomeric state of GapR, we affinity purified full-length wild-type (WT) GapR_1–89_ with no nuclease treatment and analyzed the protein by size exclusion chromatography. The protein eluted at the void volume of the Superdex 200 column under low-salt conditions (Fig. 1A), indicating mass above 600 kDa. When resolved on a native PAGE gel, the protein was found to be a heterogeneous mixture, with a regular increment of mass between two consecutive bands (Fig. 1B). This migration profile differs from the smear observed for the *Caulobacter* NAP HU (Fig. 1B), in agreement with the distinct mode by which these proteins bind DNA (3, 7).

Upon treating GapR samples with a combination of high salt and EDTA, we observed a larger right-hand shoulder of the void peak corresponding to copurified DNA and an additional elution peak far later than the void volume corresponding to GapR_1–89_ (Fig. 1A). These data suggest that GapR_1–89_ remains as a nucleoprotein complex throughout our purification procedure but dissociates into smaller units when treated with high salt and EDTA. Because units of GapR_1–89_ that are separated from copurified DNA are no longer found in the void volume after decreasing salt and removing EDTA (Fig. 1A), we reasoned that DNA may act as a platform for the formation of higher-order structures.

GapR in the presence of high salt and EDTA eluted faster than dimeric HU (Fig. 1A) (7). Using cross-linking experiments and size exclusion chromatography, both performed under high-salt and EDTA conditions to prevent association of GapR with any contaminating DNA molecules, we determined that GapR_1–89_ separated from DNA is a tetramer (Fig. 1C; see also Fig. S1A in the supplemental material). In cross-linking experiments, faint bands were observed in addition to the main band corresponding to tetrameric GapR (Fig. 1C). These faint bands may correspond to reaction products in which only two out of the four subunits were cross-linked (2.28-fold the molecular weight of the monomer) or to nonspecific cross-linking of tetramers (7.61-fold the molecular weight of the monomer) (see the table at the bottom in Fig. 1C). Alternatively, these bands could indicate the presence of GapR in oligomeric states other than tetramer, which cannot be detected by size exclusion chromatography (Fig. S1A). We determined the oligomeric state of both tagged and untagged GapR_1–89_ in low-salt buffer and observed the same results as for His_6_-GapR_1–89_ in high salt and EDTA (Fig. S1B), indicating that neither the His tag nor high salt + EDTA affects GapR oligomerization. One difference between the purification procedures that led to either dimeric (3) or tetrameric (Fig. 1C and Fig. S1A) GapR is the use of phosphate (dimer) or HEPES (tetramer) as the buffering agent. However, GapR still remained a tetramer when HEPES replaced phosphate (Fig. S1B), ruling out the possibility that phosphate ions stabilize GapR as a dimer in solution.

10.1128/mBio.00448-20.1 FIG S1 GapR is a tetramer in solution. (A) Determination of the oligomeric state of GapR proteins. Affinity-purified proteins were separated from copurified DNA and analyzed by size exclusion chromatography using a Superdex 75 10/300 GL column in the presence of 1 M NaCl and 1 mM EDTA. The protein samples were analyzed at the following concentrations: His_6_-GapR_1–89_ at 50 μM (monomer), His_6_-GapR_1–69_ at 125 μM (monomer), and His_6_-GapR_1–76_ at 100 μM (monomer). The panel below the plot lists the apparent oligomeric state of each major elution peak. (B) Effect of His tag and buffer composition on the oligomeric state of GapR_1–89_. Tagged and untagged GapR_1–89_ were dialyzed against either 20 mM HEPES-150 mM NaCl-10% glycerol or 50 mM sodium phosphate-150 mM NaCl-10% glycerol and assayed in cross-linking reactions. Proteins at 50 μM (monomer) were treated with 400 mM EDC plus 100 mM NHS for 2 h at room temperature, the reaction products were resolved by SDS-PAGE, and the gel was silver stained. As a control, proteins incubated for 2 h at room temperature in the absence of the cross-linking agents were resolved by SDS-PAGE, and the gel was stained with Coomassie blue. The panel below the gel lists the apparent oligomeric state of each major band. The figure shows that phosphate causes both tagged and untagged GapR_1–89_ to migrate slightly faster in SDS-PAGE than the proteins in HEPES buffer. Download FIG S1, TIF file, 2.5 MB. Copyright © 2020 Lourenço et al. 2020 Lourenço et al. This content is distributed under the terms of the Creative Commons Attribution 4.0 International license.

### Helix 3 drives the assembly of dimers into a tetrameric structure.

The crystal structure of DNA-bound GapR shows that H2 contacts the C-terminal region of H3 (residues A77 to I86, containing several hydrophobic residues) of another GapR subunit (Fig. 1C) (3). The segment of H3 encompassing residues R69 to D76 appears not to interact with H2 (3). To determine the contribution of the hydrophobic region of H3 to GapR oligomerization, we constructed truncated proteins lacking either the entire H3 (GapR_1–69_) or only the hydrophobic patch in this helix (GapR_1–76_). Both truncated proteins assembled primarily into dimers (Fig. 1C and Fig. S1A). The faint bands may represent nonspecific cross-linking of dimers or the ability of the truncated proteins GapR_1–69_ and GapR_1–76_ to form higher-order structures. This result argues that H3 in the C-terminal region of GapR represents a critical structural element for assembling the tetrameric protein.

### Helix 1 self-associates into a coiled-coil structure.

**FIG 2:**
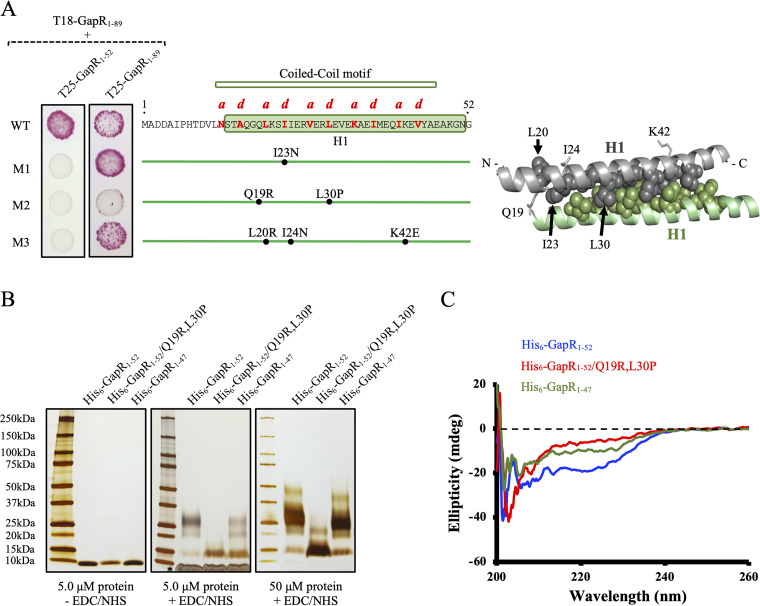
Mutants in the H1 coiled-coil motif exhibit altered folding and oligomeric states. (A) Screening for mutations that affect H1 self-association using a bacterial two-hybrid system. The left panel shows E. coli BTH101 cells expressing the T18 domain of adenylate cyclase N-terminally fused to full-length WT GapR and the T25 domain of the same enzyme fused to the truncated N terminus (GapR_1–52_ and the mutant GapR_1–52_ protein M1, M2, or M3, isolated by random mutagenesis using error-prone PCR). Fusions of the T25 domain to full-length proteins (GapR_1–89_ and the mutant GapR_1–89_ protein M1, M2, or M3) were also expressed in cells producing T18-GapR_1–89_. WT and mutant strains were grown to exponential phase (OD_600_ of 0.5), and 3 μl from each culture was spotted in 1% maltose-containing MacConkey plates. The plates were imaged after 2 days at 30°C. The predicted amino acid residues important for stabilization of canonical coiled-coil structures are denoted as “a” and “d” in the GapR sequence shown in the middle panel and are represented as spheres in the dimeric H1-H1 derived from the DNA-bound crystal structure (PDB 6CG8) (3) in the right panel. Both the middle and right panels also show the amino acid substitutions identified in the mutant alleles (residues other than those at “a” and “d” positions are indicated as sticks in the dimeric structure). GapR subunits are differentiated on the basis of color (gray and green). (B) Determination of the oligomerization state of truncated GapR proteins. Proteins at 5 and 50 μM (monomer) were cross-linked using 400 mM EDC plus 100 mM NHS for 2 h at room temperature, the reaction products were resolved by SDS-PAGE, and the gel was silver stained. Reactions were conducted in the presence of 150 mM NaCl. As a control, 5 μM (monomer) proteins incubated for 2 h at room temperature in the absence of the cross-linking agents were resolved by SDS-PAGE. The asterisk indicates a band observed in cross-linker-treated samples that had to be incubated for a long time to develop a signal, suggesting it may correspond to the cross-linker itself. (C) Circular dichroism of truncated GapR proteins at 25 μM (monomer).

It has been proposed that H1 corresponds to a second oligomerization site (3). Provided that H1 forms a coiled-coil structure (3), oligomerization would be prevented by mutations replacing residues at position “a” or “d” of the heptad repeats of the GapR coiled-coil motif (Fig. 2A, middle panel). To test this hypothesis, the coding sequence encompassing H1 (*gapR*_1–52_) was randomly mutagenized by error-prone PCR, and the amplicons were used to construct a library into a bacterial two-hybrid system. We identified three mutant *gapR*_1–52_ alleles (M1 to M3) defective in oligomerization (Fig. 2A). M1, M2, and M3 code for proteins with amino acid substitutions at position “a” or “d.” Curiously, assays with full-length GapR_1–89_ proteins containing the M1 and M3 alleles revealed no defect in oligomerization (Fig. 2A). This result suggests that the presence of the DNA binding domain on M1 and M3 GapR proteins stabilizes association between the mutant and wild-type proteins. The M2 allele containing the Q19R,L30P amino acid substitutions, however, affected GapR self-association even when the C terminus was present (Fig. 2A), indicating a more severe destabilizing effect on the protein structure compared with the mutations found in the other alleles.

Using cross-linking experiments and size exclusion chromatography, we confirmed that GapR_1–52_/Q19R,L30P is deficient in self-association compared with GapR_1–52_ (Fig. 2B and Fig. S2). Interestingly, a truncated N-terminal GapR protein (GapR_1–47_) was found to be capable of oligomerizing at both 50 and 500 μM but not at 5 μM (Fig. 2B and Fig. S2A). The main band observed for GapR_1–52_/Q19R,L30P in cross-linking experiments is slightly shifted in comparison with the protein in the control gel (Fig. 2B), as cross-linking reagents cause a mass increment by reacting with abundant acidic residues. For GapR_1–52_ and GapR_1–47_, the band at the highest intensity is about 2-fold the mass of the major band observed in the GapR_1–52_/Q19R,L30P sample (Fig. 2B). Moreover, GapR_1–52_ and GapR_1–47_ formed an intermediate band (Fig. 2B), which likely corresponds to fast-migrating dimers containing intramolecular in addition to intermolecular cross-linking. For all three proteins, bands above the major signal could represent nonspecific cross-linking or the existence of higher-order structures at very low concentrations (Fig. 2B). Circular dichroism (CD) analyses showed that both GapR_1–52_/Q19R,L30P and GapR_1–47_ have helical content lower than that determined for GapR_1–52_ (Fig. 2C). These results suggest that either trimming H1 by a few residues or introducing the Q19R,L30P substitutions into the full-length H1 reduces helical fold and increases dissociation constant of H1 self-association.

10.1128/mBio.00448-20.2 FIG S2 The Q19R,L30P substitutions alter the folding and the oligomeric state of H1. (A) Determination of the oligomerization state of truncated GapR proteins. Proteins at 500 μM (monomer) were cross-linked using 400 mM EDC plus 100 mM NHS for 2 h at room temperature, the reaction products were resolved by SDS-PAGE, and the gel was silver stained. Reactions were conducted in the presence of 150 mM NaCl. (B) Affinity-purified His-tagged proteins at the concentrations of 50 μM (monomer) (solid lines) and 1 mM (monomer) (dotted lines) were analyzed by size exclusion chromatography using a Superdex 75 10/300 GL column in the presence of 150 mM NaCl. No protein was detected on the peak denoted by ‡. The panel below the plot lists the apparent oligomeric state of each major elution peak. (C) SAXS analysis of truncated GapR proteins at 500 μM (monomer). The upper panel shows SAXS profiles of the proteins. Insets: Guinier regimes used to calculate the radius of gyration (Rg) of each protein. The panel below the plot lists the radius of gyration and molecular weight of each protein. The lower panel shows the Kratky plot, a mathematical transformation of the SAXS data. Download FIG S2, TIF file, 2.5 MB. Copyright © 2020 Lourenço et al. 2020 Lourenço et al. This content is distributed under the terms of the Creative Commons Attribution 4.0 International license.

We also carried out small-angle X-ray scattering (SAXS) measurements of GapR_1–52_ and GapR_1–52_/Q19R,L30P. Although the molecular weight calculated for GapR_1–52_ was about 2-fold that of GapR_1–52_/Q19R,L30P, Guinier analysis yielded similar radii of gyration (Rg) for the proteins (Fig. S2C). This result suggests that the spherical dimensions of the soluble protein particles created by GapR_1–52_ and GapR_1–52_/Q19R,L30P are roughly the same, despite the disruption in oligomeric state. The Kratky plot, which gives us information about flexibility and folding state (8), shows that the signal for GapR_1–52_/Q19R,L30P fails to return to baseline at high *q* values (Fig. S2C). Therefore, GapR_1–52_/Q19R,L30P is more flexible than GapR_1–52_, in agreement with a decreased folding of the mutant protein.

When the *gapR* coding sequence was replaced with the mutant Q19R,L30P allele (Fig. S3A), we observed that cell growth was compromised at both 22 and 30°C (Fig. S3B). Moreover, cells exhibited morphological defects, with a significant increase in the mean cell length and a broader distribution of sizes (Fig. S3C and Table S1), suggesting that robust oligomerization of GapR is directly linked to cellular fitness.

10.1128/mBio.00448-20.3 FIG S3 Mutations selectively disrupting oligomerization or DNA binding activity of GapR reduce cell growth and increase cell length. (A) Left panel: schematic of the strategy used to replace wild-type *gapR* for a mutant copy of the gene. Right panel: screening for *gapR* mutants. Colonies at the indicated time point after plating in the selection medium were screened. For each strain, the number of colonies analyzed and the number of mutant colonies identified are represented. (B) Growth analysis of mutant *gapR* strains. Saturated overnight cultures grown at 22°C were diluted to an OD_600_ of 0.1 and incubated at both 22 and 30°C. At the indicated time points, aliquots were taken for CFU. Values are the percentage of CFU at the indicated time points (*t* = 2, 4, and 6 h) relative to that determined for the freshly diluted cultures (*t* = 0). Results are means from three independent biological experiments, and bars indicate standard deviations. (C) Cell morphology analysis of mutant *gapR* strains. Exponentially growing cells (OD_600_ of 0.3 to 0.5) at both 22 and 30°C were imaged by phase-contrast microscopy. The length of individual cells from each strain was determined by the MicrobeJ tool (A. Ducret, E. M. Quardokus, and Y. V. Brun, Nat Microbiol 1:16077, 2016, https://doi.org/10.1038/nmicrobiol.2016.77), and data analysis was performed using the R statistical program. The numbers of individual cells used for analysis were as follows: WT *gapR*_22°C = 3,343, *gapR*/K59A_22°C = 1,226, *gapR*/R65A,K66A_22°C = 1,585, *gapR*/Q19R,L30P_22°C = 1,645, WT *gapR*_30°C = 2,378, *gapR*/K59A_30°C = 1,235, *gapR*/R65A,K66A_30°C = 1,477, *gapR*/Q19R,L30P_30°C = 690. Representative images of each strain grown at 30°C are shown. For both panels, asterisks represent statistically different values according to unpaired Student’s *t* test (*P* < 0.001). Download FIG S3, TIF file, 2.0 MB. Copyright © 2020 Lourenço et al. 2020 Lourenço et al. This content is distributed under the terms of the Creative Commons Attribution 4.0 International license.

10.1128/mBio.00448-20.8 TABLE S1 Statistical analysis of the data obtained by measuring the cell length of individual cells. Download Table S1, DOCX file, 0.01 MB. Copyright © 2020 Lourenço et al. 2020 Lourenço et al. This content is distributed under the terms of the Creative Commons Attribution 4.0 International license.

### Positively charged residues within helix 2 GapR are critical for DNA binding.

**FIG 3:**
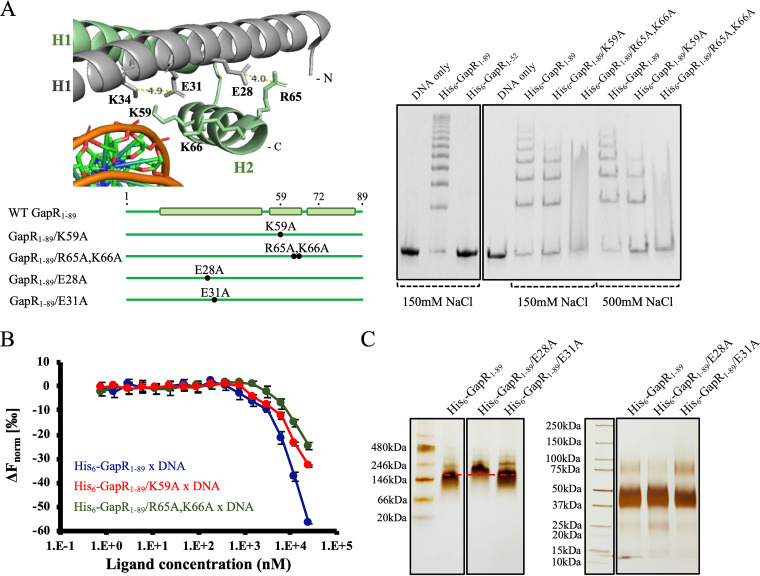
GapR binds DNA using highly conserved, positively charged residues within helix H2. (A) Analysis of the DNA binding affinity of GapR proteins. The left panel shows positively charged residues at H2 and their possible interactions in a structural representation derived from the DNA-bound crystal structure (PDB 6CG8) (3). GapR subunits are differentiated on the basis of color (gray and green). A schematic of the mutant GapR proteins constructed on the basis of the position of amino acid substitutions in H1 and H2 is shown below the structural representation. The right panel shows electrophoretic mobility shift assays of wild-type and mutant GapR proteins incubated with DNA (the promoter region of the *pilA* gene). A 2.5 μM concentration of protein (monomer) was incubated with 0.1 μM 320-bp P_pilA_ DNA for 30 min at room temperature in the presence of either 150 or 500 mM NaCl, the reaction were products were resolved by PAGE under native conditions, and the gels were stained with ethidium bromide. Controls with no protein included are also shown. (B) Microscale thermophoresis experiments with GapR proteins. An 0.1 μM concentration of ATTO 488-labeled 320-bp P_pilA_ DNA fragment was incubated with WT and mutant GapR proteins (0.8 nM to 25.0 μM) for 30 min at room temperature in the presence of 150 mM NaCl, and thermophoresis was determined for each sample. Values are changes in the normalized fluorescence upon heating (Δ*F*_norm_ = *F*_hot_/*F*_cold_) from 3 independent measurements. (C) Analysis of GapR proteins by native PAGE (left panel) and cross-linking assay (right panel). For the native PAGE, proteins at 10 μM (monomer) were used. For the cross-linking reaction, proteins at 50 μM (monomer) were treated with 400 mM EDC plus 100 mM NHS for 2 h at room temperature, and the reaction products were resolved by SDS-PAGE. The gels were silver stained. Reactions were conducted in the presence of 150 mM NaCl. The side chains of E31 and K34 from the same subunit are close to each other (panel A, left), suggesting a possible interaction. Thus, no effect on H2 is expected with the E31A mutation.

GapR was proposed to bind DNA using positively charged residues in helices H1 and H2 (3). To determine the role of these regions in DNA binding, we compared GapR_1–52_, which corresponds to H1 only, and the full-length GapR_1–89_ with respect to the ability to bind DNA. While the strong association of GapR_1–89_ with DNA accounts for the detection of the protein as part of nucleoprotein complexes in the void volume of the Superdex 200 column, GapR_1–52_ was retarded in its passage through the column, and no detectable DNA was found to copurify with the protein (Fig. S4). Furthermore, electrophoretic mobility shift assays (EMSAs) showed that GapR_1–52_ does not bind DNA, in contrast to GapR_1–89_ (Fig. 3A, right panel). Thus, our data show that H2 is necessary for DNA binding.

10.1128/mBio.00448-20.4 FIG S4 H1 of GapR is not sufficient to copurify DNA. His_6_-GapR_1–89_ and His_6_-GapR_1–52_ purified without a nuclease treatment were analyzed by size exclusion chromatography using a Superdex 200 10/300 GL column in the presence of 150 mM NaCl. A 50 μM concentration of protein (monomer) was used for each run. Fractions 8 to 20 were resolved by SDS-PAGE, and the gels were silver stained. The delayed detection of the protein by SDS-PAGE with respect to the UV trace occurs because the protein has to travel some distance after leaving the UV detector until its elution into the collection tube, as can also be seen in Fig. 1A. Inset: SDS-PAGE of the affinity-purified proteins. The gel was stained with Coomassie blue and then with ethidium bromide. Arrows indicate proteins, and the smear corresponds to copurified DNA, which is visible only in the His_6_-GapR_1–89_ sample. Download FIG S4, TIF file, 2.5 MB. Copyright © 2020 Lourenço et al. 2020 Lourenço et al. This content is distributed under the terms of the Creative Commons Attribution 4.0 International license.

K56, K59, and K66, all located in H2, are conserved residues pointed toward the DNA phosphate backbone (3). R65 is also conserved among GapR orthologs but seems to play a structural role, possibly participating in an electrostatic interaction with E28 from the other subunit in the same dimeric component of the tetramer (3). To evaluate the role of specific amino acid residues in the association of GapR with DNA, we constructed two full-length mutant proteins, GapR_1–89_/K59A, with a mutation in one of the residues possibly involved in DNA binding, and GapR_1–89_/R65A,K66A, which carries a mutation in both a putative DNA binding residue and a possible structural residue (Fig. 3A, left panel). Compared with wild-type GapR_1–89_, GapR_1–89_/K59A was clearly compromised with respect to DNA binding (Fig. 3A, right panel). Because the nucleoprotein complexes containing GapR_1–89_/R65A,K66A form a smear rather than individual bands (Fig. 3A, right panel), a precise comparison between this mutant protein and WT GapR was not possible using EMSA. To circumvent this problem, we compared the DNA binding activities of the GapR proteins using microscale thermophoresis (MST) (9). As we could not obtain GapR_1–89_/R65A,K66A at concentrations higher than 25 μM, MST experiments were carried out with all proteins up to 25 μM. Even though these protein concentrations were not sufficient to reach DNA saturation and determine the dissociation constants, we observed that the mutant proteins at any concentration ranging from 0.8 to 25 μM led to smaller changes in the normalized fluorescence of DNA molecules upon heating (Δ*F*_norm_) compared with WT GapR_1–89_ (Fig. 3B). These data imply decreased binding of the mutant proteins to DNA relative to WT GapR. Further, we showed that GapR_1–89_/R65A,K66A affected the variation of the normalized fluorescence of DNA molecules to a lesser extent than GapR_1–89_/K59A (Fig. 3B), indicating a more severe effect of substituting R65 and K66 relative to the K59A mutation. As expected, the K59A and R65A,K66A mutations had no effect on oligomerization (Fig. S5). Therefore, we showed that highly conserved, positively charged amino acid residues in H2 are important for DNA binding.

10.1128/mBio.00448-20.5 FIG S5 Mutant GapR proteins defective in DNA binding are able to oligomerize. The ability of mutant GapR proteins to interact with wild-type GapR was analyzed by bacterial two-hybrid assays. E. coli BTH101 cells expressing the T18 domain of adenylate cyclase N-terminally fused to wild-type full-length GapR and the T25 domain of the same enzyme fused to the N terminus of different mutant full-length GapR proteins were grown to exponential phase (OD_600_ of 0.5), and 3 μl from each culture was spotted in 1% maltose-containing MacConkey plates. The plates were imaged after 2 days at 30°C. Download FIG S5, TIF file, 2.5 MB. Copyright © 2020 Lourenço et al. 2020 Lourenço et al. This content is distributed under the terms of the Creative Commons Attribution 4.0 International license.

To test the hypothesis that the interaction between E28 and R65 plays a structural role (3), a full-length GapR protein with an E28A substitution was constructed (Fig. 3A, left panel). This mutation slowed the migration of the protein under native PAGE compared with WT GapR_1–89_ despite having no effect on oligomerization (Fig. 3C). The slower migration of GapR_1–89_/E28A is not caused by a change in the net charge of GapR, as an E31A mutation does not affect the electrophoretic migration of the protein (Fig. 3C). Therefore, these results are consistent with the idea that the ionic pair E28-K65 is important for the GapR structure.

We also replaced the *gapR* coding sequence with the K59 and R65A,K66A alleles (Fig. S3A) and tested the phenotype of cells bearing these amino acid substitutions. Although cells containing the K59A mutation exhibited no growth defect (Fig. S3B), they are slightly longer than wild-type cells. However, the growth of cells expressing GapR_1–89_/R65A,K66A was affected at 30°C, and a significant increase in the mean cell length was observed for this strain at both 22 and 30°C (Fig. S3C and Table S1). Thus, mutations reducing the DNA binding activity of GapR compromise cell fitness.

In agreement with the hypothesis that GapR binds DNA by contacting phosphate groups (3), we showed that the minor groove binding reagent netropsin does not compete for the binding of GapR_1–89_ to DNA (Fig. S6). Instead, the binding affinity of GapR_1–89_ for netropsin-bound DNA was found to be slightly higher than that determined for the interaction of GapR_1–89_ with DNA (Fig. S6A).

10.1128/mBio.00448-20.6 FIG S6 GapR does not compete with netropsin for binding DNA. (A) Evaluation of the effect of netropsin on the association of GapR with DNA by microscale thermophoresis. An 0.1 μM concentration of ATTO 488-labeled 12-mer DNA fragment was treated, or not, with 1.25 μM netropsin for 30 min at room temperature in the presence of 150 mM NaCl, GapR at varied concentrations (3.0 nM to 200 μM) was added, and the resulting mixtures were incubated for an additional 30 min at room temperature. DNA thermophoresis was determined for each sample. The concentration of netropsin used in the assays was determined by incubating 0.1 μM ATTO 488-labeled 12-mer DNA fragment with different amounts of netropsin (0.15 μM to 5 mM) for 30 min at room temperature and monitoring DNA thermophoresis. Because DNA saturation was reached in these experiments, the MST data were represented as the fraction of bound DNA molecules at each concentration of the ligand. Results are from 3 independent measurements. The panel below the plot lists the ligand concentrations that reach half-saturation of the fluorescently labeled molecules. (B) The DNA binding affinity of His_6_-GapR_1–89_ was monitored by electrophoretic mobility shift assays. An 0.1 μM concentration of 320-bp P_pilA_ DNA was treated with different concentrations of netropsin (10 nM to 1 mM) for 30 min at room temperature in the presence of 150 mM NaCl. A 2.5 μM concentration of His_6_-GapR_1–89_ (monomer) was added, and the resulting mixtures were incubated for an additional 30 min at room temperature. Controls with no His_6_-GapR_1–89_ included were performed to evaluate the binding of netropsin to DNA. The reaction products were resolved by PAGE under native conditions, and the gels were stained with ethidium bromide. Download FIG S6, TIF file, 2.5 MB. Copyright © 2020 Lourenço et al. 2020 Lourenço et al. This content is distributed under the terms of the Creative Commons Attribution 4.0 International license.

### GapR stimulates DNA bridging *in vitro*.

**FIG 4:**
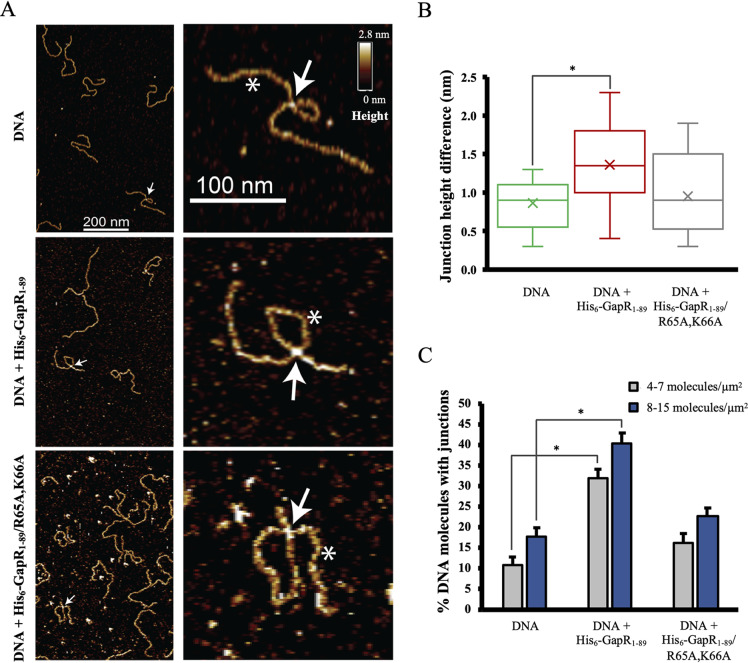
GapR binding stimulates the formation of junctions in DNA molecules. (A) Left panels show fluid atomic force microscopy scans of a 1-kbp DNA fragment with no protein added (top panel), incubated with 100 nM GapR_1–89_ (WT) (middle panel) or incubated with 100 nM GapR_1–89_/R65A,K66A (mutant defective in DNA binding) (bottom panel). Right panels show zoomed-in images of individual DNA molecules marked with the white arrow shown in the corresponding left panels. In the magnified images, overlapping regions of DNA are marked with a white arrow while well-separated DNA regions are marked using an asterisk. All AFM images are shown at the same brightness and contrast for comparison against the color map (0 to 2.8 nm). (B) Distribution of the height differences between junctions marked with a white arrow and protein-free DNA marked with an asterisk in panel A. Height differences were calculated for 17 junctions for DNA alone, 34 junctions for DNA plus GapR_1–89_, and 12 junctions for DNA plus GapR_1–89_/R65A,K66A. The junction height differences (nm), mean ± SEM, were as follows: 0.86 ± 0.08 nm (DNA with no protein added), 1.36 ± 0.08 nm (DNA plus GapR_1–89_), and 0.95 ± 0.16 nm (DNA plus GapR_1–89_/R65A,K66A). (C) Quantification of junctions observed in each sample. These measurements were performed at two different densities of molecules. Total numbers of molecules counted for each measurement are 151 (DNA alone), 219 (DNA plus GapR), and 204 (DNA plus GapR_1–89_/R65A,K66A). Asterisks in bar plots represent statistically different values according to unpaired Student’s *t* test (*P* < 0.001).

To determine the effect of GapR binding on the nanoscale organization of DNA, we visualized single nucleoprotein complexes with fluid-atomic force microscopy (AFM). Fluid-AFM provides sufficient spatial resolution to observe molecular substructures without fixing or drying samples, thus preserving their native state (10). For fluid-AFM, we used a 1-kbp DNA molecule containing a 316-bp region of the *pilA* promoter from C. crescentus. The 1-kbp DNA fragment was long enough to form random or protein-mediated loops. AFM showed well-separated single molecules matching the expected length of 1-kbp DNA (∼340 nm) (Fig. 4A, left panels). AFM images of DNA molecules in the absence of added protein revealed a low percentage of intramolecular junctions (Fig. 4A, left top panel, and Fig. 4C). However, addition of GapR_1–89_ to DNA displayed a higher frequency of intramolecular junctions than did DNA molecules alone (Fig. 4A, left middle panel, and Fig. 4C). This increase in intramolecular junctions was lost when DNA was incubated with GapR_1–89_/R65A,K66A, a DNA binding defective protein (Fig. 4A, left bottom panel, and Fig. 4C). Intermolecular junctions were observed in the DNA-alone sample at a lower frequency than the DNA-GapR_1–89_ complex upon increasing the volume added to mica, thereby increasing the sample density (Fig. S7A). AFM is capable of providing relative heights of features in a sample, which serves as a proxy for presence or absence of protein. Accordingly, we compared feature heights at both DNA junctions (white arrow) and protein-free regions (white asterisk) in the presence or absence of the WT GapR or mutant protein (Fig. 4A, right panels). Quantification of junction height differences showed that the relative increase in junction height for DNA molecules incubated with GapR_1–89_ was significantly greater than those measured for DNA molecules without added GapR_1–89_ (Fig. 4B). Although the height measurement suggests the presence of GapR_1–89_/R65A,K66A at some junctions, owing to its residual DNA binding activity (Fig. 3B), the distribution of the height differences for this mutant was similar to the random overlaps with DNA alone (Fig. 4B). Therefore, the increase in junction heights may be attributed to the association of GapR with DNA at these junctions. We asked whether GapR binds nonspecifically to DNA junctions, or if GapR affects the formation of these junctions. To answer this question, we quantified junctions in samples of DNA, DNA-GapR_1–89_, and DNA-GapR_1–89_/R65A,K66A as a percentage of total observed molecules. To rule out density-dependent effects, we performed these measurements at two different densities of molecules (Fig. 4C). We found a significantly higher percentage of junctions in DNA molecules when GapR_1–89_ was present compared with DNA molecules imaged in the absence of the protein, at both high and low molecular density (Fig. 4C). In agreement with a decreased binding affinity of GapR_1–89_/R65A,K66A for DNA (Fig. 3B) and a smaller junction height difference observed for this protein (Fig. 4B), the DNA-GapR_1–89_/R65A,K66A sample showed a percentage of junctions similar to that calculated for DNA alone (Fig. 4C). We further asked whether GapR prefers inter- or intramolecular junctions. Doubling the molecular density of the nucleoprotein complex caused a 25-fold increase in the percentage of intermolecular junctions, while the percentage of intramolecular junctions was reduced by 1.5-fold (Fig. S7B). These results suggest that GapR does not have a preference for inter- or intramolecular association *in vitro*. All experiments shown were performed with the same amount of protein (100 nM). Protein concentrations higher than 100 nM led to nonspecific binding of protein to the AFM probe and to deposition of protein on the substrate, preventing the acquisition of high-quality images. Additionally, the DNA-GapR_1–89_/R65A,K66A sample showed a large amount of protein aggregates, likely due to the reduced affinity of the protein for DNA. Despite these limitations, the AFM experiments argue that GapR stimulates inter- and intramolecular bridging of DNA *in vitro*.

10.1128/mBio.00448-20.7 FIG S7 GapR does not have a preference for inter- or intramolecular association *in vitro.* (A) Representative images of 1-kbp DNA incubated with 100 nM GapR_1–89_ and imaged using fluid AFM show molecules with intermolecular junction (green arrows) or intramolecular junction (yellow arrow). Yellow asterisk indicates likely observation of a GapR-DNA complex (assessed from heights). The termini of each molecule are marked by the numbers 1 and 1′ (for molecule 1) and 2 and 2′ (for molecule 2). Next to the prime terminus, the average measured length of the molecule is shown from five repeats of manual measurement of the molecule using a freehand line selection tool in Fiji (J. Schindelin, I. Arganda-Carreras, E. Frise, V. Kaynig, et al., Nat Methods 9:676–682, 2012, https://doi.org/10.1038/nmeth.2019). (B) Bar plot showing the percentage of molecules of DNA (or DNA plus GapR_1–89_) displaying intermolecular and intramolecular associations. These data are shown for both low (4 to 7 molecules/μm^2^) and high (8 to 15 molecules/μm^2^) densities. Asterisks represent statistically different values according to unpaired Student’s *t* test (*P* < 0.001). Download FIG S7, TIF file, 2.5 MB. Copyright © 2020 Lourenço et al. 2020 Lourenço et al. This content is distributed under the terms of the Creative Commons Attribution 4.0 International license.

### Functionally distinct and evolutionarily distant NAPs GapR and H-NS share two conserved structural elements.

**FIG 5:**
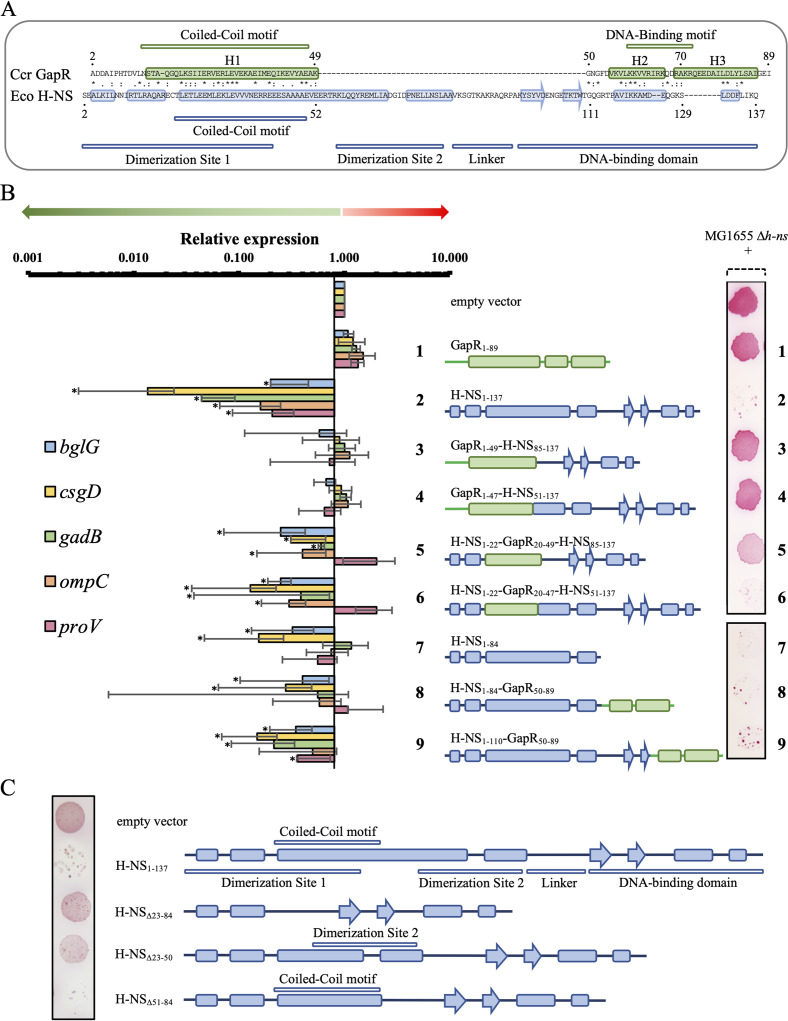
GapR and H-NS share two functionally similar regions. (A) Comparison of the deduced amino acid sequences of C. crescentus GapR and E. coli H-NS. The sequences were compared by Clustal Omega (33), and the alignment was refined manually. Structurally determined elements are shown as boxed regions for α-helices and boxed regions with arrowheads for β-sheets (shown in green for GapR and blue for H-NS). Green bars shown above the GapR sequence correspond to the previously reported coiled-coil and DNA binding motifs (2). Blue bars shown below the E. coli H-NS sequence correspond to the coiled-coil motif, the dimerization sites 1 and 2, the flexible linker, and the DNA binding domain (11–14, 21). The N-terminal methionine was omitted from both amino acid sequences in order to allow a more precise alignment. (B) Ability of chimeric proteins to repress gene expression. The right panel shows diagrams of the *Caulobacter* GapR (green), E. coli H-NS (blue), and chimera constructs, numbered 1 to 9, next to the ability of strains carrying these constructs to repress the *bgl* operon in Δ*h-ns* E. coli. MG1655 Δ*h-ns* E. coli (21) carrying derivatives of the arabinose-inducible expression vector pBAD33 was grown in the presence of 0.02% arabinose, and 3 μl from each culture was spotted on a MacConkey plate supplemented with 0.5% salicin and 0.02% arabinose, incubated at 37°C for 24 h, and imaged (34). H-NS, but not GapR, was able to repress the *bgl* operon. The left panel shows values representing the fold change in gene expression in the corresponding chimera strains compared to cells harboring the empty vector. qRT-PCR experiments were performed with total RNA extracted from cells bearing each chimera grown for 24 h in the presence of 0.02% arabinose. Results were normalized using the *rpoD* gene as an endogenous control. Data are mean values from three independent experiments; bars represent the standard deviation. Values considered statistically significant (*P* < 0.001 using unpaired Student’s *t* test) are denoted by asterisks. Color-coded bar graphs of the expression levels of 5 genes known to be repressed by WT H-NS (*bglG*, *csgD*, *gadB*, *ompC*, and *proV*) are shown for each of the chimeras. (C) Dimerization site 1 but not dimerization site 2 is critical for the ability of H-NS to repress the *bgl* operon. The *bgl* assay was performed as described above.

Comparison of GapR and H-NS sequences revealed two regions of GapR with clear similarity to H-NS (Fig. 5A). (i) The N-terminal region of GapR (residues 2 to 49), which contains H1, displays 27% identity and 61% similarity to residues 4 to 52 of H-NS. Like GapR (3), a coiled-coil motif lies within the N-terminal region of H-NS and drives oligomerization in a dimeric, antiparallel coiled-coil structure referred to as dimerization site 1 (11–13) (Fig. 5A). (ii) The segment of GapR that encompasses H2 (residues 50 to 70) (3) shares 29% identity and 67% similarity with a part of the DNA binding domain of H-NS (residues 111 to 129) (14) (Fig. 5A).

To determine the degree of functional similarity between the two regions of GapR and H-NS sharing sequence similarity, we constructed chimeras and compared them with wild-type H-NS and GapR proteins with respect to their ability to repress gene expression in an Δ*h-ns* Escherichia coli background. Expression of H-NS, but not GapR, was found to reduce transcript levels of all H-NS-dependent genes analyzed and prevent salicin utilization, which depends on the expression of the *bgl* operon (15) (Fig. 5B, constructs 1 and 2). Figure 5B shows that cells expressing chimeras in which the N-terminal region of GapR (residues 1 to 49) replaced either the H-NS dimerization sites 1 and 2 (construct 3) or just dimerization site 1 (construct 4) failed to repress transcript levels of H-NS-dependent genes and salicin utilization. However, expression of H-NS proteins containing a segment of GapR that comprises its coiled-coil motif (residues 20 to 47, H1) fused to the N-terminal region of H-NS (residues 1 to 22) to create a chimeric dimerization site 1 led to decreased expression of all but one gene (*proV*) and compromised the ability of cells to grow in the presence of salicin (Fig. 5B, constructs 5 and 6). The coiled-coil motif in dimerization site 1 is necessary for H-NS to repress expression of the *bgl* operon and prevent salicin utilization (Fig. 5C, compare H-NS_Δ23–50_ and H-NS_Δ23–84_ with H-NS_1–137_). On the other hand, dimerization site 2 was found to be dispensable for both H-NS (Fig. 5C, H-NS_Δ51–84_) and a chimera containing the coiled-coil motif of GapR (Fig. 5B, construct 5) to silence expression of the genes tested. Together, our results indicate that H1 of GapR is functionally similar to the coiled-coil of H-NS.

Repression of the *bgl* operon has been shown to occur in E. coli carrying an H-NS truncated protein in which the entire DNA binding domain is absent (16). We confirmed these data and found that *csgD* is also repressed by an H-NS protein containing only the oligomerization domain (Fig. 5B, construct 7). However, repression of *gadB* and *proV* requires full-length H-NS (Fig. 5B, left panel, construct 7). Robust repression of *gadB* and *proV* was also rescued by a chimera in which H2 and H3 of GapR (residues 50 to 89) replaced part of the DNA binding motif of H-NS (construct 9). We note that neither *gadB* nor *proV* was significantly repressed in construct 8, which has the entire H-NS C-terminal DNA binding domain replaced by H2 and H3 of GapR (Fig. 5B, left panel). Based on these results, we propose that, in addition to the shared function of their N-terminal coiled-coil motif, GapR and H-NS also share a functionally similar region in their C termini.

## DISCUSSION

### Insights into the structure and function of GapR.

In a previous study, GapR was isolated as a dimer and formed a dimer of dimers (tetramer) when incubated with DNA (3). Here, we demonstrate that GapR copurified with DNA maintains a tetrameric state upon its separation from DNA, suggesting that the presence of DNA is not an absolute requirement for the maintenance of a GapR tetramer. We were able to purify dimeric GapR only when H3 was partially or completely deleted. A study published while we were writing this paper also showed that GapR exists as a tetramer even in the absence of DNA (6). Nevertheless, DNA-free GapR tetramers, which did not yield diffraction-quality crystals (6), may be structurally different from DNA-bound tetramers, reasoning that DNA could be important for this structural change.

It was estimated that GapR is present at approximately 3,000 molecules per cell (calculated from the monomeric state) in wild-type *Caulobacter* (3). Considering that the *Caulobacter* stalked cell is roughly 1 × 10^−15^ liter (0.7 μm by 0.7 μm by 2.0 μm), GapR intracellular concentration would be 5 μM. However, only a minor fraction of these GapR molecules, perhaps at the nanomolar range or even below, is expected to be present in the dissociated state according to the distribution of GapR in *Caulobacter* (2). Therefore, even though our data imply that GapR at 50 μM remains a tetramer upon its dissociation from DNA, the actual oligomeric state of DNA-free tetramers at the physiological concentrations remains to be determined.

GapR is thought to stimulate type II topoisomerases to relax positive supercoiling in front of the replication and transcription machines (3). As GapR binds both overtwisted DNA (3) and B-DNA (6), GapR could remain associated with DNA even after the topoisomerases switch the DNA conformation from overtwisted to the relaxed state, but the movement of the replication and transcription machines would ultimately dissociate GapR as previously suggested (2).

The involvement in DNA binding of positively charged residues of the H2 helix reinforces the finding that GapR binds DNA mainly by interactions with the phosphate backbone (3). Moreover, the ability of GapR/K59A and GapR/R65A,K66A to bind DNA, despite their decreased affinities, supports the idea (3) that more than one residue is critical for stabilizing the nucleoprotein complexes. We showed here that H1 of GapR in isolation does not support DNA binding (Fig. 3A, right panel; see also Fig. S4 in the supplemental material) even though the basic residues K34, K42, and K49 were found to contact phosphates of the bound DNA molecule (3). However, it is still possible that the basic residues in H1 stabilize the H2-mediated association of the full-length protein with DNA. It may be that the reported complete loss of the DNA binding activity of a GapR mutant with basic residues in H1 (K34, K42, and K49) and H2 (K56, K59, and K66) all replaced by glutamic acid (6), is solely due to the loss of positively charged residues in H2. As we have shown here, the more severe consequences of the R65A,K66A mutation for GapR activity and cellular fitness compared with the K59A substitution are most probably the result of a combination of the absence of a residue directly contacting DNA (K66) and a residue playing a structural role (R65) in GapR/R65A,K66A. The E28-R65 pair may keep the position of H2 relative to H1, so disruption of this interaction could result in a more open conformation of the tetramer.

**FIG 6:**
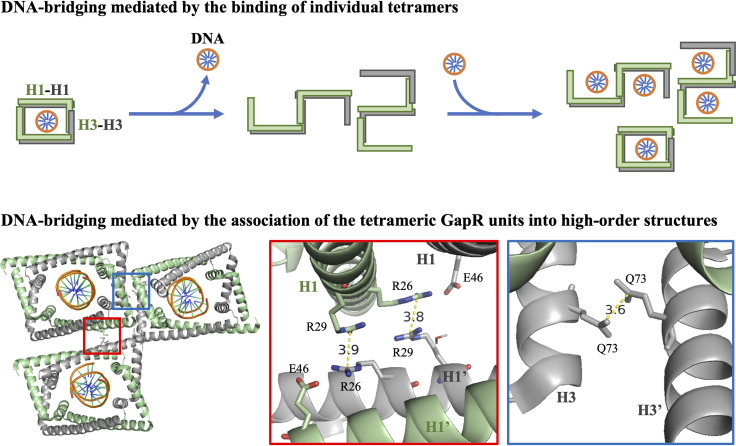
Two potential modes of GapR-mediated DNA bridging. The upper panel shows a schematic of the possible alternative conformations for GapR tetramer to illustrate the DNA-bridging model mediated by the binding of individual tetramers. H2 is omitted for simplicity. The lower panel shows three DNA-bound GapR tetramers as observed in the crystal lattice of PDB 6CG8 (3) to illustrate an alternative model in which DNA bridging is caused by the association of the tetrameric GapR units into high-order structures. Two regions that may potentially mediate DNA-bridging interactions are included as zoom insets. The prime differentiates GapR subunits in different tetramers.

We observed that GapR can mediate DNA bridging. The possibility that GapR exists as a tetramer opened at one side (6) suggests a model in which DNA bridging is promoted by individual tetramers. Rotating one monomer with respect to its partner may allow linked GapR subunits to bind more than one segment of the nucleoid simultaneously (Fig. 6, upper panel). Alternatively, self-association of DNA-bound, closed tetramers could also support DNA bridging (Fig. 6, lower panel). Inspection of the crystal structure of DNA-bound GapR (3) reveals that two arginine residues (R26 and R29) are located on the outer surface of GapR H1 (Fig. 6, lower panel). These residues in neighboring tetramers are 3.5 to 4.0 Å apart, and a salt bridge formed between R26 and E46 in the companion subunit of the same tetramer could serve to stabilize the close proximity of the arginine residues. In addition, the crystal structure also revealed one situation in which a water molecule is near R29 (3). The proximity of a water molecule could contribute to the stabilization of the interaction among the arginine residues. Therefore, R26 and R29 have the potential to form an arginine cluster (17–20). Similarly, Q73 in H3 of neighboring tetramers may form a close association (3.5 to 4.0 Å apart), offering an additional molecular interaction that may be involved in intertetramer associations (Fig. 6, lower panel).

Regardless of the mechanism that GapR uses to bridge DNA, the bridging activity could provide a physical barrier against the propagation of positive supercoils along the large spatial domains of the *Caulobacter* nucleoid, thereby facilitating the action of the type II topoisomerases. In addition to GapR, type II topoisomerases may be enriched where positive supercoils are constantly formed. Thus, by maintaining positive supercoils near the topoisomerases, GapR contributes to DNA relaxation and stimulation of the passage of the replication and transcription machines.

### The conservation of structural elements in functionally distinct NAPs.

GapR and H-NS exhibit short regions of sequence conservation and are quite different with respect to tertiary structure, oligomerization, and DNA binding (3, 6, 11–14, 21, 22). However, GapR and H-NS share two functionally similar regions. One of these regions in GapR corresponds to its H1 helix, as highlighted by a functional chimera generated by replacing the H-NS coiled-coil motif with H1 of GapR. However, residues 1 to 22 of E. coli H-NS, which precedes its coiled-coil motif and is crucial for gene silencing (12), has no counterpart in GapR. Interestingly, this short N-terminal region is degenerated in some H-NS homologs (23, 24), rendering these proteins more similar to GapR.

The H2 helix corresponds to the second region of GapR that is conserved in H-NS. A chimera generated by fusing H2 and H3 of GapR with the two β-strands of the H-NS DNA binding domain was able to silence gene expression. This finding suggests that the chimera probably has a DNA binding domain structurally and functionally similar to that found in wild-type H-NS. In the chimera, H2 of GapR may fulfill the function of the α-helix in the DNA binding domain of H-NS, as these regions display sequence similarity. H3 of GapR and the last short helix of H-NS are completely different, but H3 of GapR could still rest against the remaining DNA binding domain as the short helix of H-NS does to shelter hydrophobic clusters (both helices have hydrophobic residues). E. coli H-NS binds DNA by inserting the glutamine and arginine of a short loop (GQGR) into the minor groove of DNA (22). In the chimera, the loop has a GNGF in the corresponding position. Although the effects of Q to N and R to F have not been investigated so far, our finding suggests that the short loop of the chimera binds DNA.

In E. coli H-NS, the helix structurally similar to H2 of GapR does not contact DNA. Nonetheless, a mutation in this helix (I119T) decreased the capability of H-NS to silence gene expression (25), possibly because the position of the loop responsible for DNA binding is misplaced in the mutant protein. Strikingly, while the helix in E. coli H-NS has an indirect role in DNA binding (14), the H-NS homolog of Xylella fastidiosa uses the corresponding helix to contact DNA (24), like that of the H2 of GapR (3).

Even though we cannot rule out the possibility of a convergent evolution, the discovery that GapR and H-NS share two structurally and functionally similar regions could imply an evolutionary relationship between the proteins. Analysis of chimeras revealed that the evolutionarily conserved self-association coiled-coil region and a DNA binding region maintained function in GapR and H-NS. Nonetheless, regions of H-NS that are either deleted or structurally distinct in GapR could explain the inability of GapR to form extended filaments and to repress gene expression, and the acquisition of a unique sequence (H3) by the evolved GapR protein gave rise to the ability to form tetramers that encircle DNA (3).

## MATERIALS AND METHODS

### Bacterial strains and growth conditions.

Strains used in this work are listed in Table S2 in the supplemental material. E. coli strains were grown in Luria broth at 37°C for all experiments with the following exceptions: (i) cells were grown in MacConkey plates containing 1% maltose for analysis of *in vivo* protein-protein interaction in a bacterial two-hybrid system, (ii) MacConkey plates supplemented with 0.5% salicin were used for the *bgl* complementation assay, and (iii) protein expression was conducted at 30°C. When appropriate, the growth medium was supplemented with 0.02% l-arabinose, 50 μg/ml ampicillin, 30 μg/ml kanamycin, 20 μg/ml chloramphenicol, 12 μg/ml tetracycline, 50 μg/ml spectinomycin, and/or 30 μg/ml streptomycin. Experiments with C. crescentus were performed using CB15N derivatives grown at 22°C or 30°C in peptone-yeast extract (PYE) medium supplemented or not with 25 μg/ml spectinomycin and 5 μg/ml streptomycin. Cell growth was monitored by measuring the optical density at 600 nm (OD_600_), and aliquots were removed, serially diluted (1:10 dilution), and plated on PYE medium for counting CFU.

10.1128/mBio.00448-20.9 TABLE S2 Strains. Download Table S2, DOCX file, 0.01 MB. Copyright © 2020 Lourenço et al. 2020 Lourenço et al. This content is distributed under the terms of the Creative Commons Attribution 4.0 International license.

### Plasmid construction.

Plasmids used in this work were constructed by the Gibson assembly method (26) and are described in Table S3. Plasmids linearized with restriction enzymes were combined with PCR products generated using oligonucleotides comprising a sequence annealing to the region to be amplified and a flanking region of homology to the target vector or to another fragment when two or more DNA fragments were assembled together in the same reaction. For random mutagenesis, the *gapR* coding sequence corresponding to amino acid residues 1 to 52 was amplified by error-prone PCR as previously described (27). Site-directed mutagenesis was performed using oligonucleotides modified to include the specific amino acid substitution. Plasmids were introduced into E. coli and C. crescentus by electroporation. Sequences of the oligonucleotides are available on request.

10.1128/mBio.00448-20.10 TABLE S3 Plasmids. Download Table S3, DOCX file, 0.02 MB. Copyright © 2020 Lourenço et al. 2020 Lourenço et al. This content is distributed under the terms of the Creative Commons Attribution 4.0 International license.

### Gene replacement in C. crescentus.

pNPTS138 derivatives were constructed with the spectinomycin/streptomycin resistance gene and its promoter inserted upstream from the wild type or a mutant copy of *gapR* along with its native promoter. The fragments flanking each side of the *gapR* locus were included in order to allow the replacement of WT *gapR* in CB15N with a mutant copy of the gene and the insertion of the sequence conferring resistance to spectinomycin and streptomycin by two homologous recombination events. Mutant strains were isolated by analyzing colonies with respect to the restriction profile of PCR-amplified *gapR* followed by sequencing of the entire region using oligonucleotides annealing outside the region cloned into pNPNTS138.

### Phase-contrast microscopy.

C. crescentus cells grown to exponential phase (0.3 < OD_600_ < 0.5) were diluted to an OD_600_ of 0.1, and 1 μl was spotted on agarose pads containing M2G minimal medium. Cells were imaged by phase-contrast microscopy using an inverted microscope (DMi800; Leica) equipped with a 100× (1.4 numerical aperture [NA]) oil objective.

### RNA extraction and quantitative real-time PCR (qRT-PCR).

E. coli cultures diluted to an OD_600_ of 0.1 were grown for 24 h, and cells were harvested (16,000 × *g* for 1 min). Cell pellets were suspended in 1 ml TRIzol reagent (Thermo Fisher Scientific), and total RNA was extracted according to manufacturer’s instructions. Samples were treated with DNase I (Thermo Fisher Scientific), and their integrity was checked by agarose gel electrophoresis. A 2.5-μg amount of DNA-free RNA samples was used as the template for cDNA synthesis in the presence of 0.2 μg random hexamer primer, 1 mM deoxynucleoside triphosphate (dNTP) mix, and 200 U RevertAid reverse transcriptase (Thermo Fisher Scientific). Real-time PCR (RT-PCR) was performed using 0.4 μM (each) gene-specific oligonucleotide and 1× Fast SYBR green master mix (Applied Biosystems). Fluorescence was monitored by the 7500 fast real-time PCR system (Applied Biosystems). Oligonucleotide sequences were designed with the Primer3 software version 0.4.0 (28) and are available on request. The threshold cycle (2^−ΔΔ^*^CT^*) method (29, 30) was utilized to calculate relative expression of genes, normalized to the *rpoD* gene (31).

### Protein expression and purification.

GapR and HU were expressed in the E. coli BL21(DE3) strain by growing cells for 3 h at 30°C in the presence of 0.5 mM isopropylthiogalactoside (IPTG). Cells were harvested, washed twice in buffer A (20 mM HEPES-NaOH, pH 7.5, 150 mM NaCl, 10% glycerol), snap-frozen in liquid nitrogen, and stored at −80°C. For protein purification, cell pellets were thawed and resuspended in buffer A supplemented with 25 mM imidazole, 1 mg/ml lysozyme, and EDTA-free protease inhibitor cocktail (Santa Cruz Biotechnology). After 1-h treatment at 4°C, cells were further disrupted by sonication. Lysates were cleared by two rounds of centrifugation at 21,000 × *g*, and cleared lysates were incubated with nickel-nitrilotriacetic acid (Ni-NTA) agarose (Thermo Fisher Scientific) preequilibrated with the same buffer used for cell lysis. The agarose beads were collected and washed three times with buffer A supplemented with increasing concentration of imidazole (25, 50, 100, 200, and 500 mM). GapR and HU eluted from the beads were concentrated using an Amicon ultracentrifugal filter with a molecular weight cutoff (MWCO) of 3 kDa (Millipore), dialyzed against buffer B (20 mM HEPES-NaOH, pH 7.5, 1 M NaCl, 1 mM EDTA, 10% glycerol), and run in a Superdex 200 10/300 GL (GE Healthcare Life Sciences) size exclusion chromatography column to separate most of the copurified DNA. After purification, the proteins were concentrated, evaluated by SDS-PAGE, snap-frozen in liquid nitrogen, and stored at −80°C. The proteins were thawed and used directly or dialyzed to buffer A prior to the usage. Protein quantification was carried out using Bradford reagent (Bio-Rad). Both analytical and preparative chromatography analyses were performed using the NGC chromatography system (Bio-Rad).

For SAXS analysis, fresh GapR proteins purified by affinity and size exclusion chromatography as described above were further purified by cation exchange chromatography using the HiTrap SP HP column (GE Healthcare Life Sciences). For cation exchange chromatography, the proteins were dialyzed against buffer C (20 mM HEPES-NaOH, pH 7.5, 50 mM NaCl, 10% glycerol), and elution was carried out using 50% buffer D (20 mM HEPES-NaOH, pH 7.5, 1 M NaCl, 10% glycerol). The proteins were then dialyzed against buffer A, quantified, and used directly for SAXS measurements.

### Microscale thermophoresis analysis.

For the thermophoresis assays, a fluorescently labeled double-strand DNA was PCR amplified or obtained by hybridization. In each case, an oligonucleotide covalently bound at its 5′ end to the ATTO-488 fluorophore was used. The hybridization was carried out by heating the oligonucleotides at 95°C for 3 min followed by incubation at 55°C for 3 min. Labeled DNA (0.1 μM) was mixed with protein in 20 mM HEPES-NaOH (pH 7.5), 150 mM NaCl, 10% glycerol, 0.05% Tween 20, and the resulting mixture was serially diluted (1:1 dilution) in the same buffer supplemented with fluorescently labeled DNA. After a 30-min incubation at room temperature, samples were analyzed in the Monolith NT.115 instrument (Nano Temper Technologies). The fluorescence was monitored for 5 s before heating, for 30 s under constant heating (LED power 80% and MST power 40), and for 5 s after deactivating the infrared laser. A 25-s delay was allowed between successive measurements. Data analysis was carried out using the software MO Affinity Analysis version 2.2.4 (Nano Temper Technologies).

### Electrophoretic mobility shift assay and native PAGE.

The electrophoretic mobility of proteins under native conditions and the electrophoretic mobility shift of DNA upon incubation with proteins were determined using gel electrophoresis on 4 to 15% polyacrylamide gels (Bio-Rad). Protein samples and loading buffer (20 mM HEPES-NaOH, pH 7.5, 150 mM NaCl, 30% glycerol, 0.05% bromophenol blue) were mixed at a ratio of 1:1 (vol/vol) and run at 4°C in 1× Tris-glycine buffer (25 mM Tris-Cl, pH 8.3, 192 mM glycine). For electrophoretic mobility shift assays, PCR-amplified DNA was incubated with protein for 30 min at room temperature in 20 mM HEPES-NaOH, pH 7.5, 10% glycerol supplemented with 150 or 500 mM NaCl and then subjected to electrophoresis as described above.

### Protein cross-linking.

Cross-linking reactions were performed by incubating proteins with 400 mM 1-ethyl-3-(-3-dimethylaminopropyl) carbodiimide hydrochloride (EDC) and 100 mM *N*-hydroxysulfosuccinimide (NHS) for 2 h in 20 mM HEPES-NaOH, pH 7.5, 150 mM NaCl, 10% glycerol. Cross-linking reactions were stopped by adding 150 mM β-mercaptoethanol, 0.1% SDS and heating at 95°C for 5 min. Samples were then mixed with SDS loading buffer, boiled for 5 min, and resolved on 4 to 15% polyacrylamide gels (Bio-Rad) under denaturing condition (25 mM Tris-Cl, pH 8.3, 192 mM glycine, 0.1% SDS).

### SAXS measurement.

Small-angle X-ray scattering (SAXS) experiments were performed at the bio-SAXS beamline BL4-2 at the Stanford Synchrotron Radiation Light source. Data were collected using a Pilatus3 X 1M detector (Dectris AG) with a 3.5-m sample-to-detector distance and beam energy of 11 keV (wavelength, λ = 1.127 Å). SAXS data were measured in the range of 0.0033 Å^−1^ ≤ *q* ≤ 0.27 Å^−1^ [*q *= 4πsin(θ)/λ, with 2θ being the scattering angle]. The *q* scale was calibrated with silver behenate powder. The GapR samples were injected directly into a temperature-controlled flow cell. The SAXS data were taken in a series of 12 1-s exposures. These images were then analyzed for possible effects of radiation damage, normalized according to the transmitted intensity, and averaged using the program SasTool.

### CD spectroscopy.

Circular dichroism (CD) measurements were performed using a J-815 circular dichroism spectrometer (Jasco). Far-UV spectra (200 to 250 nm) were recorded in a 1-mm-path-length cell with an exposure time of 1 s/nm. The sample cell was maintained at 15°C, and three scans were collected and averaged for each sample. A buffer spectrum was subtracted from all sample spectra before plotting.

### Atomic force microscopy.

DNA molecules used for AFM experiments were composed of the *pilA* promoter (316 bp) and its downstream region (704 bp) and were obtained by amplification using PCRs. DNA was incubated alone or in the presence of GapR (wild-type and a mutant protein) for 16 h at 4°C and then imaged on a BioScope Resolve Bio AFM (Bruker). The long incubation time was used to ensure that the binding reaction reached the equilibrium. The microscope itself was placed in an isolation box to minimize drift, temperature fluctuations, and vibrations. Rapid force-distance (PeakForce tapping mode) imaging modality was used to obtain images. We used a high-resolution AFM probe (PeakForce HIRS-F-B, spring constant *k* = 0.12 N/m) for imaging. After deploying the probe on the AFM head, a laser alignment was performed followed by a thermal tuning calibration of the probe in MilliQ water. For high-resolution AFM of DNA and DNA-GapR complexes, we used freshly cleaved mica as a substrate. Mica was cleaved by removing five to six layers using scotch tape. To minimize sample drift, we affixed cleaved mica on steel specimen discs (Ted Pella, Inc.) using optical glue (NOA68; Norland optical adhesives). The optical glue also created a hydrophobic barrier around the mica, thus creating a sample well that minimized sample overflow and evaporation. The sample chamber was then mounted on the microscope using magnetic mounts. Wet wipes were kept around the chamber and AFM head to further minimize evaporation. One hundred microliters of imaging buffer containing 20 mM HEPES-NaOH (pH 7.5), 150 mM NaCl, 10% glycerol, 10 mM NiCl_2_ was added to the sample chamber. Next, 20 μl of sample (DNA or DNA plus GapR) was added to the imaging chamber. After addition of the sample, we pipetted 20 μl of the imaging solution up and down five times to ensure uniform mixing. This procedure led to a very reproducible sample density across different fields of view. After mixing the sample, the aligned AFM head was carefully placed on the microscope and the cantilever was moved down until it was submerged in the sample. At this stage, another round of laser-guided calibration and thermal tune calibration was performed. Finally, the AFM cantilever was engaged with the sample, and we waited for 5 to 20 min before changing any parameters to equilibrate the system and improve stability. We scanned a 1-μm by 1-μm region to obtain multiple DNA molecules in one field of view. Nanoscope software was used for data acquisition. Typically, we acquired 15 to 20 images with the high-resolution tip before observing a deterioration in the data quality. Due to this limitation, we used a new tip for each sample, keeping all the scanning parameters the same. For analyzing AFM data, we developed a workflow that comprised a first round of image processing (image flattening using a zero-, first-, and second-order polynomial applied to each scan line) followed by particle segmentation using sample heights as a cutoff. The cutoff was selected such that we were able to threshold >90% of DNA molecules in the image. Image processing and segmentation were performed in Bruker NanoScope Analysis software v 9.0. Postsegmentation, we obtained heights of DNA (or DNA plus GapR) segments and exported them into text files that were processed using bespoke programs written in Matlab (MathWorks).
