# Supplementary material for: The Nucleoid-Associated Protein GapR Uses Conserved Structural Elements To Oligomerize and Bind DNA
Source: mBio. 2020 Jun 9;11(3):e00448-20. doi: 10.1128/mBio.00448-20 (PMC7373187; doi:10.1128/mBio.00448-20)
Supplement: TABLE S1 [file mBio.00448-20-st001.docx]

**Table S1.** Statistical analysis of the data obtained by measuring the cell length of individual cells.

| **Mean length (μm) of *C. crescentus* cells expressing different GapR proteins** | | | | | | | | | |
| --- | --- | --- | --- | --- | --- | --- | --- | --- | --- |
| **Temperature (°C)** | | **WT *gapR*** | | ***gapR*/K59A** | ***gapR*/R65A,K66A** | ***gapR*/Q19R,L30P** | | | |
| 22°C | | 3.148 | | 3.786 | 4.355 | 5.177 | | | |
| 30°C | | 3.216 | | 3.872 | 5.091 | 5.282 | | | |
| **Comparison of the cell length distribution of different strains at the same temperature** | | | | | | | | | |
|  | | **Temperature (°C)** | | ***gapR*/K59A x WT** | ***gapR*/R65A,K66A x WT** | ***gapR*/Q19R,L30P x WT** | | | |
| mean length difference (μm) (IC - 95%) | | 22°C | | 0.639  (0.564 to 0.713) | 1.207  (1.119 to 1.295) | 2.029  (1.897 to 2.161) | | | |
|  |  | 30°C | | 0.656  (0.563 to 0.749) | 1.875  (1.761 to 1.989) | 2.066  (1.828 to 2.304) | | | |
| **Comparison of the cell length distribution of each strain at different temperatures** | | | | | | | | | |
|  | **WT**  **(22 x 30°C)** | | ***gapR*/K59A**  **(22 x 30°C)** | | ***gapR*/R65A,K66A**  **(22 x 30°C)** | | | ***gapR*/Q19R,L30P**  **(22 x 30°C)** |  |
| mean length difference (μm) (IC - 95%) | | 0.068  (0.018 to 0.118) | 0.085  (-0.023 to 0.193) | | 0.736  (0.601 to 0.871) | | 0.106  (-0.161 to 0.373) | |  |
