# Supplementary material for: The Nucleoid-Associated Protein GapR Uses Conserved Structural Elements To Oligomerize and Bind DNA
Source: mBio. 2020 Jun 9;11(3):e00448-20. doi: 10.1128/mBio.00448-20 (PMC7373187; doi:10.1128/mBio.00448-20)
Supplement: TABLE S2 [file mBio.00448-20-st002.docx]

**Table S2.** Strains

| **Strain** | **Description** | **Source** |
| --- | --- | --- |
| ***E. coli*** |  |  |
| NEB Turbo | *F’ proA^+^B^+^ lacI^q^ ΔlacZM15/fhuA2 Δ(lac-proAB) glnV galK16 galE15 R(zgb-210::Tn10)Tet^S^ endA1 thi-1 Δ(hsdS-mcrB)5*; for cloning | New England Biolabs |
| BL21 (DE3) | fhuA2 [lon] ompT gal (λ DE3) [dcm] ∆hsdS λ DE3 = λ sBamHIo ∆EcoRI-B int::(lacI::PlacUV5::T7 gene1) i21 ∆nin5; for protein expression | New England Biolabs |
| ∆*hns* | MG1655 *hns*::*tetRA*; for *bgl* assay | ^a^ |
| BTH101 | *F^-^ cya-99 araD139 galE15 galK16 rpsL1 hsdR2 mcrA1 mcrB1*; for bacterial two-hybrid assay | Euromedex |
| ***C. crescentus*** |  |  |
| NA1000 | Holdfast mutant derivative of wild-type CB15; for phenotypic analyses | ^b^ |
| *gapR*/Q19R,L30P | NA1000 *gapR*/Q19R,L30P, Spec^R^; for phenotypic analyses | This work |
| *gapR*/K59A | NA1000 *gapR*/K59A, Spec^R^; for phenotypic analyses | This work |
| *gapR*/R65A,K66A | NA1000 *gapR*/R65A,K66A, Spec^R^; for phenotypic analyses | This work |

^a^ Y. Gao, Y. H. Foo, R. S. Winardhi, Q. Tang, J. Yan, and L. J. Kenney, Proc Natl Acad Sci U S A 114:12560-12565, 2017, https://doi.org/10.1073/pnas.1716721114.

^b^ M. Evinger, and N. Agabian, J Bacteriol 132:294-301, 1977.
