# Supplementary material for: The Nucleoid-Associated Protein GapR Uses Conserved Structural Elements To Oligomerize and Bind DNA
Source: mBio. 2020 Jun 9;11(3):e00448-20. doi: 10.1128/mBio.00448-20 (PMC7373187; doi:10.1128/mBio.00448-20)
Supplement: TABLE S3 [file mBio.00448-20-st003.docx]

**Table S3.** Plasmids

| **Plasmid** | **Description** | **Source** |
| --- | --- | --- |
| pBAD33 | Replicating vector for arabinose-inducible expression; Cm^R^ | ^a^ |
| pKT25 | Replicating vector for IPTG-inducible expression of CyaA^T25^ fused to the N terminus of a protein of interest; Kan^R^ | Euromedex |
| pUT18C | Replicating vector for PTG-inducible expression of CyaA^T18^ fused to the N terminus of a protein of interest; Amp^R^ | Euromedex |
| pET28b | Replicating vector for IPTG-inducible expression of His_6_ fused to the N terminus of a protein of interest; Kan^R^ | Novagen |
| pNPTS138 | Suicide vector for two-step recombination; Kan^R^, Suc^S^ | ^b^ |
| pBAD33*gapR*1-89 | For arabinose-inducible expression of GapR_1-89_ | ^c^ |
| pBAD33*hns*1-137 | For arabinose-inducible expression of H-NS_1-137_ | ^c^ |
| pBAD33*gapR*1-49-*hns*85-137 | For arabinose-inducible expression of GapR_1-49_-H-NS_85-137_ | This work |
| pBAD33*gapR*1-47-*hns*51-137 | For arabinose-inducible expression of GapR_1-47_-H-NS_51-137_ | This work |
| pBAD33*hns*1-22-*gapR*20-49-*hns*85-137 | For arabinose-inducible expression of H-NS_1-22_-GapR_20-49_-H-NS_85-137_ | This work |
| pBAD33*hns*1-22-*gapR*20-47-*hns*51-137 | For arabinose-inducible expression of H-NS_1-22_-GapR_20-47_-H-NS_51-137_ | This work |
| pBAD33*hns*1-84 | For arabinose-inducible expression of H-NS_1-84_ | This work |
| pBAD33*hns*1-84-*gapR*50-89 | For arabinose-inducible expression of H-NS_1-84_-GapR_50-89_ | This work |
| pBAD33*hns*1-110-*gapR*50-89 | For arabinose-inducible expression of H-NS_1-110_-GapR_50-89_ | This work |
| pBAD33*hns*Δ23-84 | For arabinose-inducible expression of H-NS with an internal deletion corresponding to residues 23-84 | This work |
| pBAD33*hns*Δ23-50 | For arabinose-inducible expression of H-NS with an internal deletion corresponding to residues 23-50 | This work |
| pBAD33*hns*Δ51-84 | For arabinose-inducible expression of H-NS with an internal deletion corresponding to residues 51-84 | This work |
| pUT18C*gapR*1-89 | For IPTG-inducible expression of CyaA^T18^-GapR_1-89_ | ^c^ |
| pKT25*gapR*1-89 | For IPTG-inducible expression of CyaA^T25^-GapR_1-89_ | ^c^ |
| pKT25*gapR*1-89/I23N (M1) | For IPTG-inducible expression of CyaA^T25^-GapR_1-89_/I23N | This work |
| pKT25*gapR*1-89/Q19R,L30P (M2) | For IPTG-inducible expression of CyaA^T25^-GapR_1-89_/Q19R,L30P | This work |
| pKT25*gapR*1-89/L20R,I24N,K42E (M3) | For IPTG-inducible expression of CyaA^T25^-GapR_1-89_/L20R,I24N,K42E | This work |
| pKT25*gapR*1-89/K59A | For IPTG-inducible expression of CyaA^T25^-GapR_1-89_/K59A | This work |
| pKT25*gapR*1-89/R65A,K66A | For IPTG-inducible expression of CyaA^T25^-GapR_1-89_/R65A,K66A | This work |
| pKT25*gapR*1-52 | For IPTG-inducible expression of CyaA^T25^-GapR_1-52_ | This work |
| pKT25*gapR*1-52/I23N (M1) | For IPTG-inducible expression of CyaA^T25^-GapR_1-52_/I23N | This work |
| pKT25*gapR*1-52/Q19R,L30P (M2) | For IPTG-inducible expression of CyaA^T25^-GapR_1-52_/Q19R,L30P | This work |
| pKT25*gapR*1-52/L20R,I24N,K42E (M3) | For IPTG-inducible expression of CyaA^T25^-GapR_1-52_/L20R,I24N,K42E | This work |
| pET28b*gapR*1-89 | For IPTG-inducible expression of His_6_-GapR_1-89_ | This work |
| pET28b*gapR*1-89/Q19R,L30P | For IPTG-inducible expression of His_6_-GapR_1-89_/Q19R,L30P | This work |
| pET28b*gapR*1-89/K59A | For IPTG-inducible expression of His_6_-GapR_1-89_/K59A | This work |
| pET28b*gapR*1-89/R65A,K66A | For IPTG-inducible expression of His_6_-GapR_1-89_/R65A,K66A | This work |
| pET28b*gapR*1-89/E28A | For IPTG-inducible expression of His_6_-GapR_1-89_/E28A | This work |
| pET28b*gapR*1-89/E31A | For IPTG-inducible expression of His_6_-GapR_1-89_/E31A | This work |
| pET28b*gapR*1-76 | For IPTG-inducible expression of His_6_-GapR_1-76_ | This work |
| pET28b*gapR*1-69 | For IPTG-inducible expression of His_6_-GapR_1-69_ | This work |
| pET28b*gapR*1-52 | For IPTG-inducible expression of His_6_-GapR_1-52_ | This work |
| pET28b*gapR*1-52/Q19R,L30P | For IPTG-inducible expression of His_6_-GapR_1-52_/Q19R,L30P | This work |
| pET28b*gapR*1-47 | For IPTG-inducible expression of His_6_-GapR_1-47_ | This work |
| pET28b*hu* | For IPTG-inducible expression of His_6_-HU | This work |
| pNPTS138*specgapR*/Q19R,L30P | For replacement of WT *gapR* with the mutant allele *gapR*/Q19R,L30P and insertion of the Ω cassette in NA1000 | This work |
| pNPTS138*specgapR*/K59A | For replacement of WT *gapR* with the mutant allele *gapR*/K59A and insertion of the Ω cassette in NA1000 | This work |
| pNPTS138*specgapR*/R65A,K66A | For replacement of WT *gapR* with the mutant allele *gapR*/R65A,K66A and insertion of the Ω cassette in NA1000 | This work |

^a^ L. M. Guzman, D. Belin, M. J. Carson, and J. Beckwith, J Bacteriol 177:4121-4130, 1995, https://doi.org/10.1128/jb.177.14.4121-4130.1995.

^b^ J. L. Ried, and A. Collmer, Gene 57:239-246, 1987, https://doi.org/10.1016/0378-1119(87)90127-2.

^c^ D. P. Ricci, M. D. Melfi, K. Lasker, D. L. Dill, H. H. McAdams, and L. Shapiro, Proc Natl Acad Sci U S A 113:E5952-E5961, 2016, https://doi.org/10.1073/pnas.1612579113.
